# Contemporary Strategies in the Management of Hepatocellular Carcinoma

**DOI:** 10.1155/2012/154056

**Published:** 2012-11-04

**Authors:** Shirin Elizabeth Khorsandi, Nigel Heaton

**Affiliations:** Institute of Liver Studies, King's College Hospital, Denmark Hill, London SE5 9RS, UK

## Abstract

Liver transplantation is the treatment of choice for selected patients with hepatocellular carcinoma (HCC) on a background of chronic liver disease. Liver resection or locoregional ablative therapies may be indicated for patients with preserved synthetic function without significant portal hypertension. Milan criteria were introduced to select suitable patients for liver transplant with low risk of tumor recurrence and 5-year survival in excess of 70%. Currently the incidence of HCC is climbing rapidly and in a current climate of organ shortage has led to the re-evaluation of locoregional therapies and resectional surgery to manage the case load. The introduction of biological therapies has had a new dimension to care, adding to the complexities of multidisciplinary team working in the management of HCC. The aim of this paper is to give a brief overview of present day management strategies and decision making.

## 1. Introduction

Hepatocellular carcinoma (HCC) is the fifth most common cancer in the world. Ninety percent of primary liver cancers are HCC, the majority of which develop on the background of cirrhosis. Over the past decade, medical management of the patient with chronic liver disease has improved. In parallel, the prevalence of hepatitis B (HBV), hepatitis C virus (HCV), alcohol related liver disease, and NASH has increased and combined with an ageing population has led to a surge in the number of cases worldwide [1–3]. As a consequence, HCC is an important complication of cirrhosis and a leading indication for liver transplantation (LT), accounting for approximately a third of patients on transplant waiting lists [4]. The introduction of surveillance using alphafetoprotein and ultrasound has led to the earlier recognition of HCC and increases the therapeutic options available [5]. In the absence of treatment the overall 5-year survival is <10% [6]. These include LT, resection, locoregional, and systemic therapies. For a solitary HCC with preserved liver function and low hepatic vein pressure gradient, liver resection still remains the first choice. 

Historically, survival rates were 35–62% at 3 years and 17–50% at 5 years for patients with cirrhosis undergoing resection for HCC [6, 7]. However, tumor recurrence rates were high, up to 70%, and progression to liver failure was common [6, 8–10]. LT is an attractive treatment option as it treats both the cancer and underlying liver disease. In the 1980s, patients presenting with large HCC were considered good candidates for LT as they were in better condition than patients with chronic liver disease and were more likely to survive the perioperative period but they had large or multifocal tumors. This resulted in a high recurrence rate of up to 65%, a 5 year survival of 10–35%, and a median survival of those with recurrence of 6 months, coupled with increasing demand for donor livers led to a more restrictive selection process [11–13]. Recognition that small tumors appeared to fare better after LT led Mazzaferro to introduce the Milan criteria to select patients leading to improved survival with low rates of tumor recurrence [14]. By adhering to the Milan criteria of undertaking LT for HCC with a solitary tumor ≤5 cm or 3 tumors ≤3 cm each, a 5 year survival greater than 70% and recurrence rates of <10% were produced. 

## 2. Staging

**Figure 1:**
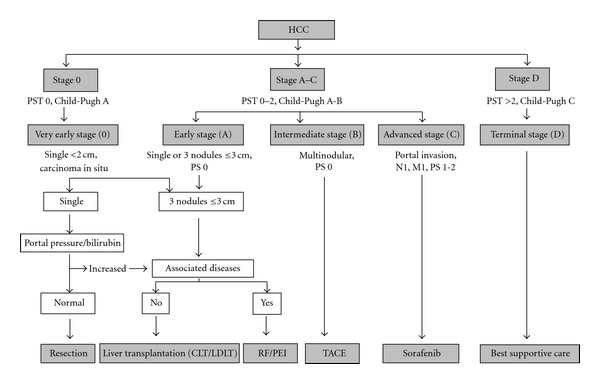
The Barcelona-Clínic Liver Cancer (BCLC) staging system for HCC. M: metastasis classification; N: node classification; PST: performance status; RFA: radiofrequency ablation; TACE: transarterial chemoembolization.

A staging system for HCC poses problems because the presence of liver disease and tumor varies due to the different epidemiological backgrounds and risk factors. The ideal staging system needs to include prognostic information regarding both the cancer and liver functional status and take account of clinical factors that influence response to treatment. The TNM classification is an oncology standard useful in conjunction with the presence of microvascular invasion of examined resection or explanted tumors/liver and provides information regarding the risk of tumor recurrence but does not take account of the liver functional status. As TNM requires pathological data (microvascular invasion) and only 20% HCC are resected for which it is good at discriminating stages for, its usability is limited. The Okuda staging system has been widely used since 1985. It uses four criteria of ascites, albumin, bilirubin, and tumor size to assess liver functional status and tumor stage. It is a good system for stratifying advanced/symptomatic disease but less useful in early stage to guide treatment choices. Other available systems are the French classification, the Cancer of the Liver Italian Program (CLIP) classification, and the Barcelona-Clínic Liver Cancer (BCLC) staging system; the Chinese University Prognostic Index (CUPI score) and the Japan Integrated Staging (JIS) or bm-JIS if biomarkers are included [15]. The CUPI and CLIP scores mainly stratify patients at advanced stages; only two include prognostic variables (BCLC, CUPI) and only one allocates treatment according to specific prognostic subclasses (BCLC). The BCLC is emerging as the standard staging system for HCC in the West and has been externally validated and incorporates prognostic variables related to tumor (size, number, vascular invasion, N1, M1), liver function (Child-Pugh), and health status (ECOG-Eastern Cooperative Oncology Group Performance Status). As well as incorporating variables that influence therapy such as bilirubin, portal hypertension, and presence of symptoms to assist in treatment decision making (see Figure 1).

## 3. Liver Transplant

LT is the treatment of choice for small multifocal HCC ≤3 tumors and ≤3 cm or a single tumor ≤5 cm with significant liver functional impairment. Better selection of patients has improved 5 year survival to >70% and recurrence <10%. The major limitation to LT as treatment for HCC is the scarcity of cadaveric donors and the associated waiting time that results in a 20% drop-out rate and potentially increases the risk of recurrence from extension of vascular invasion. The use of tumor size and number to try to reflect tumor biology has been successful. However, it is clear that some patients with favourable “biology” are excluded. A number of groups have tried to expand indications beyond the Milan criteria and claim to achieve similar survival rates [16, 17]. The University of California San Francisco (UCSF) criteria are probably the best known and include one tumor ≤6.5 cm or multiple tumors of which the largest is 4.5 cm and the sum of all diameters is ≤8 cm [16]. More recently the up-to-7 criteria, where the HCC scores 7 based on the sum of the largest tumor (diameter cm) and the total number of tumors, have been introduced [17]. The majority of the studies supporting extension of the Milan Criteria are based on retrospective histological analysis of the tumor burden in the explant liver and have not been validated prospectively [16–19]. 

Another area where the principles of the Milan Criteria have been challenged is in salvage LT. Salvage LT has been advocated by some to manage HCC within Milan Criteria after resection [20]. In selected cases, similar overall 5 year survival for salvage LT as primary LT for HCC has been achieved (provided the comparison is from time listed for LT rather than date of LT, that is, intention to treat bias). There is continuing debate regarding whether previous resection compromises the subsequent LT [21]. Other groups have found salvage LT to have a high operative mortality, 23.5% versus 2.1% for primary LT, higher recurrence rates, and poorer overall 5 year survival of 41% [22]. Salvage LT remains controversial at a time of a limited resource with tumor characteristics, background liver (cirrhotic or noncirrhotic), and centre experience appearing to be the main determinants of recurrence and survival. 

An alternative strategy to expanding the criteria for LT is to downstage to within Milan Criteria aiming to achieve patient survival and recurrence free survival rates similar to those treated at an earlier stage. This is distinct from bridging therapy. Bridging therapy is utilised to maintain the tumor within listing criteria while a suitable graft is awaited for on the waiting list. Bridging therapy is a widely accepted practice whereas downstaging for LT is not [23, 24]. To be eligible for downstaging locoregional therapy, there should be no radiological evidence of vascular invasion. There is no consensus limit to tumor number or size [25]. Predictors of downstaging failure are tumors with an infiltrative pattern [26] and an AFP >1000 ng/mL [27]. There is evidence to suggest that downstaging of HCC to within Milan Criteria can produce reasonable results [25, 27]. But the data is difficult to interpret as the studies utilise different inclusion criteria (tumor size and number), locoregional therapies either individually or in combination, and endpoints. Current published data reveal that after downstaging, surgical resection rates vary widely between 7% and 18% producing 5 year survival rates of between 25% and 57% [28] and LT rates range between 24% and 90%, with an intention to treat post HCC treatment survival of between 60% and 70% at 3 years [27, 29, 30]. 

Living donor LT (LDLT) is a good graft option for HCC as it allows neoadjuvant treatment to be organized around a LT. It provides a high-quality graft and removes a competing HCC recipient from the waiting list. But higher recurrence rates and reduced survival have been reported when compared to cadaveric LT [31–33]. Explanations for this observation include growth factors released from the regenerating liver may stimulate cancer cell growth. The shorter waiting time for LDLT may remove the observation period that occurs on the waiting list to assess tumor biology and a 3 month cooling off period has been advocated before undertaking LDLT. Surgical oncological clearance may also be compromised as the IVC has to be preserved for LDLT [34]. In addition, an element of institutional bias may lead to LDLT in HCC with a higher risk of recurrence. On multivariate analysis of published studies on LDLT versus cadaveric LT, graft type, and waiting time have not been found to be significant risk factors for recurrence post LT. If LDLT is undertaken for HCC outside regional criteria and the graft fails retransplantation with cadaveric LT is ethically contentious [35]. The donor risk and the degree of benefit to the recipient needed to justify LDLT for advanced HCC are still undetermined and for now many centres have adopted the same criteria/therapeutic goals for LDLT as cadaveric LT [36].

## 4. Liver Resection

Resection is the treatment of choice in noncirrhotics. Noncirrhotic HCC accounts for 5% of cases in the West and 40% of cases in Asia. Patients with cirrhosis suitable for resection need preserved liver function and a hepatic venous pressure gradient ≤10 mmHg. Anatomic resection is advocated by some as being more preferable to nonanatomic, as it is thought to produce better outcome by eliminating intrahepatic metastases in the related portal vein tributary [37]. In patients with cirrhosis selected on liver functional status, the main predictors for survival are tumor size, multiplicity, and vascular invasion. Five-year survival for tumors ≤2 cm, 2–5 cm, and >5 cm are 66%, 52%, and 37%, respectively. For single tumors the 5 year survival is 57% and for multiple 26% but some centres are achieving >50% in multiple HCC within the Milan Criteria but otherwise are not suitable for LT [38]. Recurrence remains problematic occurring in 70% at 5 years; true recurrence/intrahepatic metastases generally occur within 2 years of resection; if greater than 2 years it is generally regarded as a de novo tumor or late recurrence [39]. At present there is no evidence that neoadjuvant/adjuvant therapy has any efficacy in reducing recurrence after resection [40]. Downstaging locoregional therapy can be employed to facilitate resection in disease which is initially regarded as unresectable and can achieve reasonable outcome, with 5 year survivals of 25–67%, with the possibility of cure [28]. Preoperative portal vein embolisation can be employed to increase future remnant liver volume to allow more extensive resections to be undertaken but the complication rate in cirrhotics is 10–20% and its effectiveness in this patient group is not fully established [41]. A laparoscopic approach to resection in cirrhotics has been proposed by some to reduce the operative insult and the risk of decompensation [42]. 

## 5. Locoregional Therapy: Ablation, TACE, and Radiation

There are a number of different locoregional strategies available or being developed but the largest experience is with transarterial chemoembolization (TACE) and radiofrequency ablation (RFA). Percutaneous ethanol injection (PEI) was the first chemical ablative technique utilised. When applied to small tumors <2 cm PEI produces 90% necroses and a 5-year survival of 47–53% but is limited by high recurrence rates of approximately 40%. Chemical ablation has now been superseded by thermal techniques such as radiofrequency ablation (RFA). RFA is the most well-studied alternative to PEI producing better local tumor control with a 2 year recurrence of 2–18% and a 5 year survival of 40–70% or better when the treatment groups have been selected [43]. Meta-analyses of randomised control trials have confirmed that RFA is a more effective way to obtain local tumor control and survival benefit compared to PEI, establishing it as a standard locoregional treatment [44, 45]. 

RFA can be performed percutaneously, laparoscopically, or at open surgery depending on tumor location [46, 47]. RFA is effective for early small HCC <3 cm when resection or LT are not feasible [48–51], whereas larger tumors may be inadequately treated. Overall 10–25% of tumors will not be suitable for RFA because of location such as subcapsular, adjacent to the gall bladder or major vessels which increases the risk of complication and inadequate ablation because of heat sink. The recurrence rate after RFA for selected early small HCC can be comparable to that of surgery [50, 51]. In highly selected HCC < 2 cm RFA has the potential to be curative with a rate of complete response approaching 97% and a 5 year survival of 68%. However, randomised control trials of RFA against resection for small HCC < 3 cm have failed to show that RFA is as effective as resection but the majority of studies were underpowered or had incomplete follow up [49, 52]. Increasingly, RFA is being considered as an alternative initial “curative” treatment option for small centrally placed HCC as it offers the advantages of preserving parenchyma, potentially removes competition from the transplant waiting list and based on location, effective tumor necrosis can be obtained [43, 48].

Solitary HCC >3 cm but <5 cm RFA becomes less effective. But when TACE followed by RFA for this size tumor is applied, the therapeutic effect of RFA is significantly increased and reduces tumor progression rate to 6% compared to 39% for RFA alone [53]. In larger HCC >5 cm outside LT criteria or not suitable for resection, ablative strategies may not work in a predictable manner. TACE also has inconsistent results and no advantage has been demonstrated by combining therapies [54]. When RFA is not suitable either because of tumor location or size, novel thermal or nonthermal ablative techniques may overcome the limits of RFA. Promising thermal ablative strategies include microwave producing large areas of ablation with less heat sink and high intensity focused US (HIFU) that can be used in patients with ascites. Alternative non thermal ablative techniques of interest include irreversible electroporation (IE). Ablative technology is improving and further experience will determine its applicability. In assessing the effectiveness of ablation radiologically, the widely used RECIST (the response evaluation criteria in solid tumours) has limitations as it includes both necrotic and viable tumor areas [55] and the modified RECIST that includes the assessment of viable tumor showing uptake in the arterial phase is more reliable. 

Based on a meta-analysis TACE is emerging as the standard of care for asymptomatic HCC outside Milan criteria [56], demonstrating improved survival compared to best supportive care. A partial response of 15–55% can be observed producing a survival benefit, increasing median survival time from 16 months to 20 months with 49% survival at 2 years [56]. But individually the studies did not clearly demonstrate a benefit, mainly because of heterogenous patient study groups and varying TACE techniques. This implies that good results with TACE are achieved when it is used on a selective basis. Generally TACE is not suitable in decompensated liver disease where there is ascites or jaundice to the avoid major complications and minimize treatment related deaths to less than 2% [57]. For optimal results TACE needs to be as selective as possible, producing sustained and high localised concentrations within the tumor minimizing systemic exposure. Alternative ways to be delivering chemotherapy instead of the standard ethiodized oil (lipiodol) suspensions are drug-eluting beads [58]. In the PRECISION V trial [59], a randomized control trial, comparing drug-eluting beads with doxorubicin to conventional TACE with doxorubicin found it was better tolerated, with reduced liver toxicity and improved treatment response. Owing to the improved safety and tolerance drug-eluting beads could be applicable in higher risk patient groups. Further ways of optimising the therapeutic benefit of TACE is by combining with systemic drugs. Using agents that target the angiogenic pathways that are switched on by the local hypoxia produced by TACE is being evaluated [45]. Generally, if there is no response after two TACE sessions, alternative treatment strategies should be considered, which in the majority will be systemic therapy. In highly selected patients consideration should be given to combination of treatments such as ablation/radioembolization.

Advanced HCC that is symptomatic, exhibiting vascular invasion and/or has extrahepatic disease have a short median survival of 6 months with 25% surviving a year [60]. Systemics are often the only treatment option for palliation but there is a subset that benefit from locoregional therapy such as where vascular invasion is limited to a venous branch receiving intra-arterial therapies such as TACE [61] or radioembolization [62]. Radioembolization using yttrium-90 (90Y) labelled microspheres a beta emitter, appears promising, and may also be effective as a precursor to radical therapy with outcomes similar to TACE [30, 63] and Sorafenib [64]. There is a need to be aware of intestinal and lung shunting which may provoke serious complications. There is a minimal embolic effect so when there is main portal vein involvement and TACE is contraindicated radioembolization with yttrium may be a good option. In the absence of portal vein involvement radioembolization in Child A survival is 15.5 months, Child B is 13 months, with a portal vein involvement survival of both Child A + B being 5.6 months [65] with 25–50% response rates [64]. 

Cyberknife is a new stereotactic body radiation therapy (SBRT) or stereotactic ablative radiotherapy (SABR) in combination with a robotic system that tracks the tumor during respiration and is able to deliver high dose radiation accurately sparing adjacent normal tissue in a small number of fractions. A number of studies in HCC not suitable for standard locoregional treatment or resection have reported promising results. In HCC <100 ml progression free survival rates at 6 months, 1 year, and 3 years of 83%, 72%, and 68% respectively, with overall survival at 1 year, and 3 years of 92.9% and 58.6% have been reported. It also has utility as local salvage treatment after TACE achieving local control in 95% [66]. To this date no serious SBRT related toxicities being reported [67–69] but it is not clear whether it can be applied to patients with more severe liver diseases as its threshold for tolerance is not defined.

## 6. Systemic Therapies: Antiviral Therapy, ****Immunosuppression, Biologicals,**** and Chemotherapy 

Worldwide 78% of HCC are viral related with 53% attributed to HBV and 25% to HCV [70]. Risk of HCC recurrence after treatment is increased with progression of active hepatitis and fibrosis. Antiviral therapy is used as an adjuvant treatment with the aim to reduce viral load and fibrosis with the aim of halting progression of viral induced liver disease and reducing the risk of further HCC developing. Hepatitis B (HBV) infection increases the risk of HCC recurrence particularly for the patient who is HBeAg + and/or has a high serum HBV DNA level [71, 72]. Treatment with nucleoside analogues entecavir or tenofovir suppresses HBV DNA levels improving liver function in decompensated liver disease and may reduce the risk of HCC development over time [73]. HBV antiviral therapy reduces risk of recurrence by 41% and overall mortality by 73%, mainly because death from liver failure is reduced by 77% [74]. Use of longterm lamivudine treatment resistance can occur in 70% over 5 years has given way to alternative nucleoside analogues such as entecavir, telbivudine, and nucleotide analogues such as tenofovir but longterm data is lacking for their effect on reducing HCC recurrence. 

Chronic infection with HCV appears to increase the rate of HCC development in a similar way to HBV. The risk does not change with genotype (G) but a recent meta-analysis suggests that HCV G1b maybe more at risk of HCC transformation [75]. Meta-analysis of adjuvant alpha IFN shows reduction in HCC recurrence and mortality in curatively ablated viral hepatitis related HCC. Individually these studies report no effect. Antiviral potency and the ability to produce a sustained viral response (SVR) in HCV appear to be associated with reducing the risk of HCC recurrence [76]. More longterm data is needed from the newer protease inhibitors (boceprevir, telaprevir) to determine whether the higher SVR they are able to produce translates into lower risk of HCC longterm.

Immunosuppressive agents compound malignant behaviour as immune surveillance for cancer cells is impaired. Mammalian target of rapamycin (mTOR) inhibitors, for example, Sirolimus is an exception to this rule. mTOR is overexpressed in approximately 2/3 of HCC making it an attractive therapeutic target [77]. To establish whether the immunosuppressive regime affects recurrence rates, data from the SiLVER Study is awaited. The SiLVER Study is a randomised multicenter clinical trial comparing Sirolimus containing to a mTOR inhibitor free immunosuppressive regimes. The study consists of a 3-year enrolment period and a 5-year followup. At present, there is little evidence on whether the immunosuppression regime should be completely changed to a mTOR inhibitor or whether these agents should be added to the preexisting immunosuppressive regime when recurrent HCC presents post LT [35, 78, 79]. CNI exposure should be minimised as there is evidence that this reduces the risk of tumor recurrence long term [80].

HCC is an unique chemoresistant tumor and until 2007 no systemic drug was recommended for its management. In the early 1990s, a number of randomised controlled studies assessed the role of adjuvant chemotherapy but no benefit or efficacy was demonstrated. Multiple agents have been assessed but doxorubicin, an anthracycline, has been most rigorously studied [81, 82]. The main patient groups that should be considered for adjuvant chemotherapy are those being transplanted for extended criteria or have a high risk of recurrence based on the explant pathology. As patients selected for LT should be at low risk of recurrence the majority will not gain any benefit from routine adjuvant chemotherapy.

Since 2007 Sorafenib, an oral tyrosine kinase inhibitor, has become the standard of care. Based upon the SHARP study that demonstrated Sorafenib improved median survival from 7.9 months to 10.7 months and slowed time to progression from 2.8 to 5.5 months [83]. It is well tolerated with diarrhoea in 8-9% and hand-foot skin reaction in 8–16%, with side effects leading to its discontinuation in 15%. Sorafenib is regarded as the standard therapy for metastatic disease and for HCC progressing despite optimal locoregional therapy [84]. A number of ongoing studies are establishing Sorafenib's adjuvant role in resection, local ablation (Sorafenib as Adjuvant Treatment in the Prevention of Recurrence of Hepatocellular Carcinoma (STORM)), and TACE. Additionally, a phase 1 study is being undertaken in high-risk patients post-LT that on explant are outside Milan criteria with microvascular or macrovascular invasion or histologically poorly differentiated HCC. At present there is no evidence of increased toxicity in LT recipients and Sorafenib can produce a response based on published case reports [85, 86]. Other biological agents entering phase 2 or 3 trials for HCC include EGFR (erlotinib) and VEGFR/FGFR (brivanib) tyrosine kinase inhibitors [87].

## 7. Conclusions

Management of HCC continues to evolve and interventional radiology in the form of TACE ± RFA increasingly dominates management either as a bridge to LT or to downstage facilitating LT or resection. As locoregional therapy technology advances patients that can be considered either for palliation or potential cure will increase. Criteria for LT listing need to become more sophisticated by incorporating tumor biology in decision making, presently inferred from clinical behaviour but in the future by the use of molecular markers. This will facilitate stratification and individualization of HCC treatment. Ultimately, the aim of LT, irrespective of disease etiology is to give the maximum benefit from a limited organ pool.
